# Ocean acidification increases cadmium accumulation in marine bivalves: a potential threat to seafood safety

**DOI:** 10.1038/srep20197

**Published:** 2016-01-21

**Authors:** Wei Shi, Xinguo Zhao, Yu Han, Zhumei Che, Xueliang Chai, Guangxu Liu

**Affiliations:** 1 College of Animal Sciences, Zhejiang University, Hangzhou, 310058, P.R. China; 2 Marine Monitoring and Forecasting Center of Zhejiang, Hangzhou, 310007, P.R. China; 3 Zhejiang Mariculture Research Institute, Wenzhou, 325005, P.R. China

## Abstract

To date, the effects of ocean acidification on toxic metals accumulation and the underlying molecular mechanism remains unknown in marine bivalve species. In the present study, the effects of the realistic future ocean *p*CO_2_ levels on the cadmium (Cd) accumulation in the gills, mantle and adductor muscles of three bivalve species, *Mytilus edulis*, *Tegillarca granosa*, and *Meretrix meretrix,* were investigated. The results obtained suggested that all species tested accumulated significantly higher Cd (*p* < 0.05) in the CO_2_ acidified seawater during the 30 days experiment and the health risk of Cd (based on the estimated target hazard quotients, THQ) via consumption of *M. meretrix* at pH 7.8 and 7.4 significantly increased 1.21 and 1.32 times respectively, suggesting a potential threat to seafood safety. The ocean acidification-induced increase in Cd accumulation may have occurred due to (i) the ocean acidification increased the concentration of Cd and the Cd^2+^/Ca^2+^ in the seawater, which in turn increased the Cd influx through Ca channel; (ii) the acidified seawater may have brought about epithelia damage, resulting in easier Cd penetration; and (iii) ocean acidification hampered Cd exclusion.

Ocean acidification occurs as a result of pumping enormous amount of carbon dioxide into the atmosphere, and it is changing seawater chemistry at an unprecedented rate. Many marine organisms are sensitive to these changes, with evidence suggesting that mass extinctions and ‘reef gaps’ were driven by ocean acidification during the Paleocene-Eocene Thermal Maximum^1^. The phenomenon has drawn much attention, with numerous effects on marine organisms have been reported^2^,^3^. Ocean acidification may negatively affect marine organisms by reducing the calcium carbonate (CaCO_3_) state^4^ and disturbing the acid-base physiology^5^ leading to reductions in the calcification rate of many shell-forming marine organisms^3^,^4^,^6^. In addition to calcification, dissolved CO_2_ may negatively affect marine organisms in numerous ways, including fertilization success^7^, embryonic development^2^, metabolism^8^, immune response^9^, and survival rate^10^.

Cadmium (Cd), which is obtained as a by-product of zinc, is a toxic metal used in a wide array of applications. A large employment of Cd in industrial and agricultural activities has led to substantial anthropogenic emissions of Cd into the marine environment. Compared with other toxic metals, Cd is relatively soluble and can be accumulated by organisms such as bivalves. These characteristics of Cd would result in toxic metal poisoning, therefore consequently, Cd is considered to be a significant environmental threat^11^. Numerous studies have shown that Cd pose adverse impacts on the immune^12^ and reproductive systems^13^ of various species, giving rise to chromosomal damage^14^.

Marine bivalves, as filter feeders, are able to concentrate pollutants to several orders of magnitude above ambient levels and accumulate toxic metals in their tissues in proportion to the amount of toxic metals in the environment. Therefore, marine bivalves are deemed to be suitable bioindicators for toxic metal pollution monitoring due to their readily interpretable biological consequences of contamination. On the contrary, many marine bivalves are traditional aquaculture species that are widely distributed throughout the world, and provide approximately 1 × 10^8^ tons seafood for consumers yearly^15^. Since toxic metals such as Cd can be accumulated by bivalves, there is an increasing concern of the bivalve safety as seafood. Nowadays, several approaches have been proposed to assess the potential health risks of toxic metals intake. Among which, the target hazard quotient (THQ, the ratio between the estimated dose of a contaminant and the reference dose) has been widely used in health risk assessment of toxic metals in food^16^. Surveys conducted in the major seafood markets of the Pearl River Delta, south China, presented an view of the Cd contamination in edible marine bivalves as a potential hazard to public health^17^. Hence, the analysis of Cd accumulation in marine bivalves can provide useful information for both marine environmental assessments and seafood safety.

Previous studies have demonstrated that changes in seawater pH and chemistry would affect the speciation, adsorption, toxicity and rates of redox processes of metals in seawater^18^. Unlike a decrease in water pH by adding a strong acid or acid rain, ocean acidification driven by increased *p*CO_2_ contains more dissolved CO_2_, HCO_3_^−^, and CO_3_^2−^ at the same pH values; therefore, more physiological processes of the organisms would be affected^11^. For instance, the acidity manipulated by CO_2_ showed a stronger toxicity to the embryonic development of sea bream^19^ and coastal meiobenthic copepods^11^. Although the effects of increased acidity on metal accumulation has been well studied, especially in freshwater organisms^20^,^21^, to our knowledge, the impacts of CO_2_-driven ocean acidification on the toxic metal accumulation in marine bivalves and the underlying mechanism remains elusive. Limited comparable studies in molluscs were conducted with cephalopods, suggesting that the increase of seawater *p*CO_2_ enhanced the uptake of toxic metal during the early life stage of these species^22^,^23^,^24^.

According to previous studies, the P-glycoproteins (PGP) have been closely linked to Cd exclusion. PGPs belong to ATP-binding cassette transporters that resist drugs and toxins by an ATP-consuming process^25^. Gene *pgp-5* is induced upon exposure to toxic metals and is reported to function as an ATP-dependent efflux pump that protects animals by exporting exogenous toxins^26^.

The present study was therefore conducted to (i) determine the effects of ocean acidification on the cadmium accumulation in gills, mantle, and adductor muscles of three aquiculture bivalves, blue mussel (*Mytilus edulis*), blood clam (*Tegillarca granosa*), and hard clam (*Meretrix meretrix*); (ii) investigate the effects of ocean acidification on the Cd^2+^/Ca^2+^ content in the water environment; (iii) evaluate the influences of ocean acidification on the expressions of Cd exclusion related *pgp-5* gene and (iv) provide firsthand evidence estimating the effects of realistic future ocean acidification on seafood safety.

## Results

At an experimental temperature of 25.5 ± 1.7 °C, the measured values of pHs for the control and two experimental trials were 8.07 ± 0.05, 7.79 ± 0.06 and 7.42 ± 0.03, respectively. Both the Cd^2+^ and Ca^2+^ concentrations in the seawater significantly differed among the three *p*CO_2_ trials (Table 1). The concentration of the cadmium and calcium increased and decreased markedly (*p* < 0.05) as the pH declined, respectively. Compared to control, significant higher Cd^2+^/Ca^2+^ ratios were found in the two CO_2_ acidified seawater samples at pH 7.79 and 7.42 (about 1.15 and 1.36 times of the control, respectively).

### Toxic metal accumulation in the various tissues of the three bivalves

**Figure 1:**
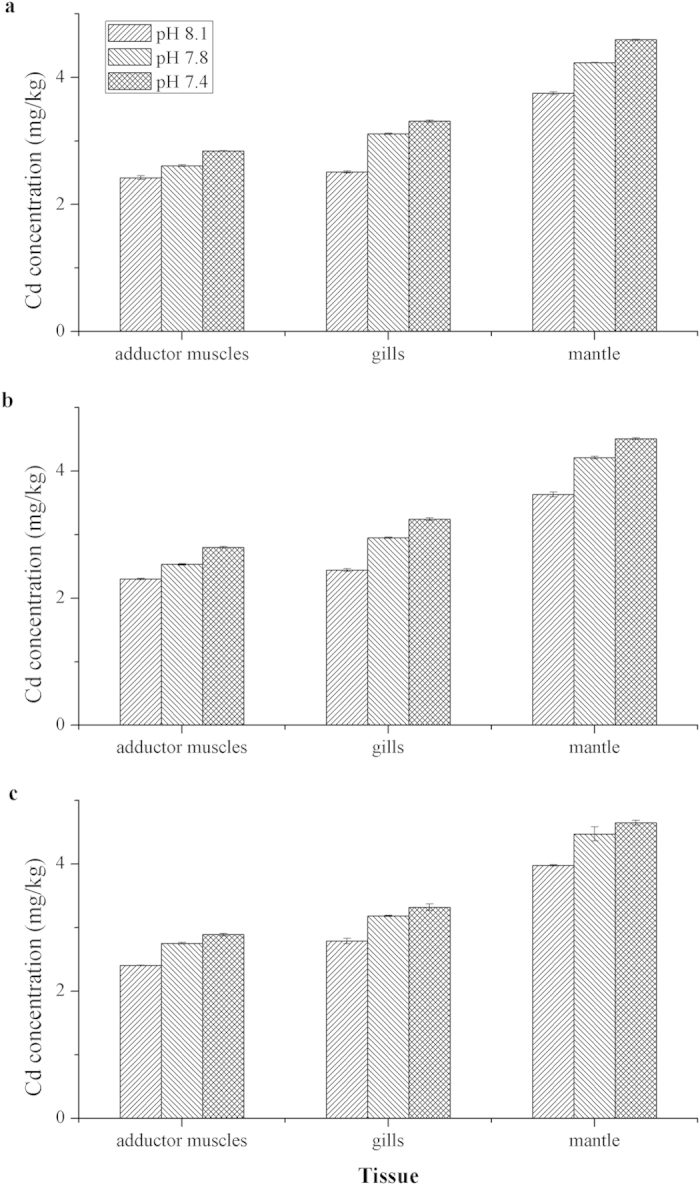
Cd concentration (mean ± SD) (mg/kg, dry weight) of different tissues of (a) *M. meretrix*, (b) *T. granosa*, and (c) *M. edulis* in different pCO_2_ trials.

After raising in seawater containing 0.05 mg/L Cd at various *p*CO_2_ levels for 30 days, the Cd accumulations in the gills, mantle and adductor muscles of *M. edulis*, *T. granosa*, and *M. meretrix* were shown in Fig. 1. Compared to control, CO_2_ acidified seawaters led to a significant higher Cd accumulation in the tissues of all three bivalves investigated. The highest Cd contents were found in the individuals from the pH 7.4 experimental groups followed by those from pH 7.8 and pH 8.1 groups. Furthermore, results obtained in the present study showed that different tissues accumulate Cd differently, with average concentrations of Cd detected were in the order of mantle > gills > adductor muscles (Fig. 1).

### Gene expressions of pgp-5 in different pCO_2_ levels

**Figure 2:**
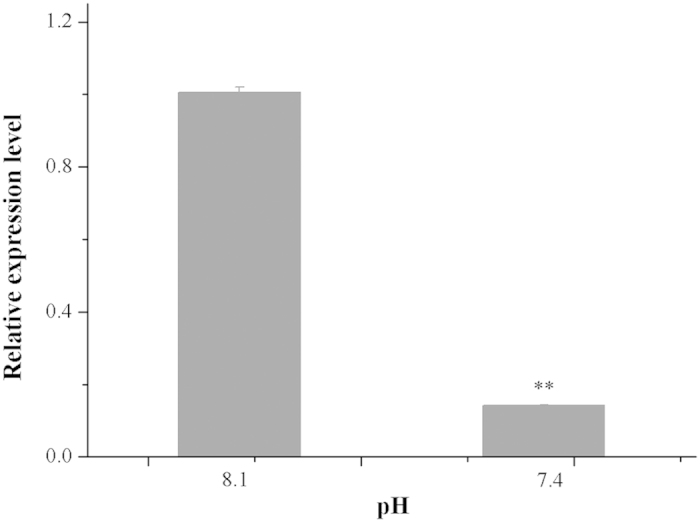
Relative expression levels (mean ± SE) of gene *pgp-5* in response to acidified seawater. (** indicate an extreme significant difference compared to that of the control by the *t*-test)

After raising in CO_2_ acidified seawater (pH 7.4) for 30 days, *pgp-5* expression was significantly lower than that of the control (*p* < 0.05). Gene expression of pgp-5 in pH 7.4 CO_2_ acidified seawater was decreased to approximately one seventh of the control (Fig. 2).

### Cd THQs of bivalves in different pCO_2_ levels

The Cd EDIs and THQs via consuming *M. meretrix* under different *p*CO_2_ levels were shown in Table 2. The THQ values of Cd were significantly higher (*p* < 0.05) when the animals were exposed to *p*CO_2_ acidified seawater, which were about 1.21 and 1.32 times of that of the control for experimental groups at pH 7.8 and 7.4, respectively.

## Discussion

**Figure 3:**
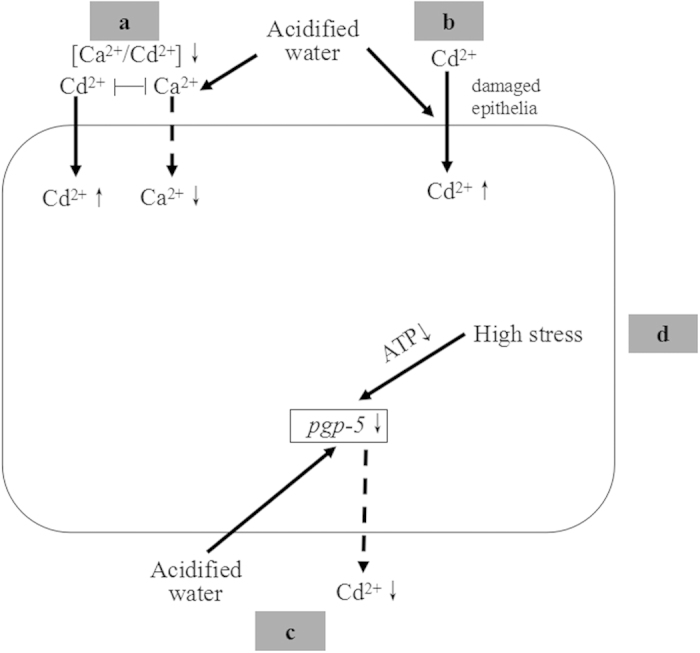
Effects of ocean acidification on cadmium uptake and exclusion. (**a)** Acidified seawater has higher Cd concentration and Cd^2+^/Ca^2+^ ratio, which facilitate the entry of Cd^2+^ through Ca^2+^ channel. (**b)** Epithelia damage as a result of acidified seawater made it more penetrable to Cd. **(c)** Acidified seawater had an inhibitory effect on the gene expression of *pgp-5*, which reduced the exclusion of Cd. (**d)** Ocean acidification may cause stress on marine organisms and constrain the energy available for Cd exclusion. For more details, see the discussion text.

Toxic metal accumulation was shown to increase with a decrease in pH upon manipulation with the addition of a strong acid^20^,^27^. Results obtained in the present study showed that ocean acidification exert a similar effect on Cd accumulation in bivalves, which might be explained by the following reasons (Fig. 3).

First, seawater acidification driven by CO_2_ may change the chemistry of toxic metal compounds and subsequently lead to an increase in the toxic metal accumulation. In the present study, the concentration of the cadmium increased markedly (*p* < 0.05) in the acidified seawater (Table 1), a finding consistent with previous studies^28^. A higher environmental Cd^2+^ contamination would be expected to facilitate Cd entry into bivalves. It was found that Cd enters the cells of mammalian^29^ and marine organisms^30^,^31^ primarily through voltage-sensitive calcium channels since the Ca^2+^ channels may fail to distinguish between Cd^2+^ and Ca^2+^ ions due to the same charge and comparable sizes. In support of this view, decreased Cd^2+^ influx was observed with the application of Ca^2+^ channel blockers (nimodipine and verapamil) in a mangrove crab *Ucides cordatus*^32^ and freshwater teleost *Oncorhynchus mykiss*^33^. Similar results were observed in *Crassostrea virginica*^34^ and *M. edulis*^35^ as well, conforming that the Ca^2+^ channel is the uptake route for Cd in marine bivalves. In addition, probably due to the competition for the same channels, Ca^2+^ was found to protect against Cd^2+^ uptake in rainbow trout (*O. mykiss*)^36^, molluscs (*Littorina littorea*)^37^ and crabs (*Carcinus maenas*)^38^. In the present study, the Ca^2+^ concentration was found to be significantly lower in the CO_2_ acidified seawater, where the concentration of Cd^2+^ and Cd^2+^/Ca^2+^ ratio were significantly higher (Table 1). Therefore, an increased extracellular Cd^2+^ concentration and a reduced inhibitory effect of Ca^2+^, have provided a favourable environment for Cd^2+^ uptake into the body of bivalves in the CO_2_ acidified seawater.

Second, acidified seawater may bring about direct damages to the bivalves tissues and subsequently affect the accumulation of toxic metals. Entry through an apical epithelial membrane is the first step in toxic metal absorption, therefore damaged epithelia will increase the penetration of metals into cells^39^. In addition, it has been shown in *M. edulis* that the decrease in the pH of seawater significantly reduced the lysosome health, as measured by the Neutral Red Retention assay^9^. Therefore, the reduction in lysosome health caused by acidification would disrupt cellular pathways and increase membrane fragility, and this may subsequently lead to the increase in Cd uptake due to a weakened defence system^40^.

Third, the changes in toxic metal exclusion could also have contributed to the higher Cd accumulation from the CO_2_ acidified seawater. The gene *pgp-5*, which belongs to the ATP-binding cassette transporters, was reported to be evoked by toxic metal stress^25^,^26^. Kurz found that *pgp-5* was induced to at least threefold by the exposure to cadmium in *Caenorhabditis elegans*^26^, which suggested that *pgp-5* was essential for a substantial resistance to Cd. As a result, a down-regulation of *pgp-5* would lead to a reduced ability to export Cd.

The exclusion of intracellular Cd by *pgp-5* is an energy consuming process and therefore is subjected to energy availability. A compensation hypothesis suggests that animals would alter energetic trade-offs among different aspects of the physiological maintenance budget to meet the increased energetic demands under stressful conditions^41^. For example, it had been shown that *M. edulis* was able to protect their tissues against seawater acidification in energetic costs, which led to a reduction in the energy budget for growth, shell formation, and toxicant metabolism^42^. Previous study has also shown that acidified seawater would suppress the expression of genes related to the tricarboxylic acid cycle, electron transport chain, and oxidative phosphorylation triggering a decrease in the production of ATP^43^. Therefore, the reduction in energy availability for Cd exportation may constrain the exclusion of intracellular Cd as well.

The potential impacts of climate change on the different aspects of human and animal health and welfare are widely debated topics. It was suggested that climate change will affect all four pillars of food security, namely, food availability, access to food, stability of food supplies and food utilization^44^. However, the potential consequences of ocean acidification on food safety of marine bivalves were largely overlooked. Marine bivalves provide an important and economical protein source for human consumption and are a primary protein source for over one billion of the poorest people in the world^15^. Moreover, marine bivalves are important food sources for supplying essential elements and are rich sources of certain vitamins, such as vitamins B_6_ and B_12_. However, since marine bivalves is capable of accumulating a large amount of pollutants, such as toxic metals in their tissues, in extreme conditions, these contaminants in the edible parts of marine bivalves can pose a severe threat to human health.

According to the data obtained in the present study, ocean acidification would increase Cd accumulation in bivalves through increased uptake and reduced exclusion. Since the intake of Cd via consuming bivalves is only part of the total oral Cd intake, a significant increase (*p* < 0.05, ANOVA) of the THQ value from less than 1/5 to about 1/4 indicated a higher risk for consumers, although all the THQ values obtained in the present study were less than the critical value of 1 implying a low risk of non-carcinogenic effects. In particular, past studies have suggested that the health risk regarding Cd contamination is increasing due to consumption of other food, such as vegetables and fruits^16^. In addition, since cultured bivalves in farms are exposed to the environmental Cd contamination for a markedly longer period (at least a year for the three species investigated) than the 30 days duration of the present study, the health risk posed by marine bivalves consumptions under future ocean acidification scenario is expected to be more severe. Furthermore, with the increased risk of toxic metal contamination brought about by ocean acidification, it is highly likely that the sea areas suitable for bivalve aquaculture and capture will shrink significantly hence reducing the overall seafood supplies.

## Methods

### Collection and acclimation of bivalves

Adult *T. granosa* (9.5 ± 1.4 g), *M. edulis* (28.0 ± 5.2 g), and *M. meretrix* (50.0 ± 8.1 g) were collected from Yueqing, Wenzhou, China in August 2014. After cleaning off the epizoa, bivalve individuals were acclimatized in a 1000 L plastic tank at an ambient water temperature of 26 ± 3 °C and pH 8.07 ± 0.05 with flowing sand filtered seawater. The sample were fed with microalgae (*Tetraselmis chui*) at the satiation feed rate daily for 7 days prior to experiment.

### Seawater acidification

The sand-filtered seawater used in the experiment was obtained from Qingjiang Bay, Zhejiang Province (28°28′N and 121°11′E) with pH at 8.07 ± 0.05, salinity at 20 ± 0.5‰ and the average background Cd concentration of 9.8 ± 0.2 μg/L. During the experiment, the bivalves were maintained under manipulated *p*CO_2_ conditions, with one ambient group at pH 8.1 (current concentration of *p*CO_2_) as the control and two experimental groups at pH 7.8 and 7.4 representing the pH values predicted by the Intergovernmental Panel on Climate Change (IPCC) to occur at 2100 and 2300, respectively^3^. The desired pH values were achieved by continuous aeration with ambient air or air-CO_2_ mixture into the filtered seawater in 60-L plastic tanks. The air-CO_2_ mixture was obtained by mixing dry CO_2_-free air and pure CO_2_ gas at known flow rates using flow controllers. The pH of each experimental trial was verified daily with a portable pH metre (Sartorius PB-10) to ensure there was no substantial pH change during the course of experiment.

### Cadmium accumulation assay

The experiments were performed using analytical grade salts of Cd (NO_3_)_2_·4H_2_O. Stock solutions were prepared in deionized water at 1 M, a concentration high enough to prevent weighing errors and salinity fluctuation. The experimental Cd concentration (0.05 mg/L) was chosen on the basis of the reported safe concentrations of these bivalve species^45^,^46^,^47^.

After one week of acclimation, the bivalves (40 *T. granosa*, 15 *M. edulis* and 20 *M. meretrix*) were randomly assigned to plastic tanks with a total seawater volume of 20 L containing approximately 0.05 mg/L Cd and maintained in the three desired *p*CO_2_ conditions. Bivalves were fed with *T. chui* and the seawater was replaced daily with pH pre-adjusted seawater to maintain the desired *p*CO_2_ level. After seawater replacement, Cd was added to achieve the designed experimental Cd concentration in the water column. The Cd accumulation assay was conducted for a 30day duration.

### Metal concentration analysis

Seawater samples were collected from each experimental trial every ten days to determine the effect of the CO_2_-driven acidification on the concentration of Cd^2+^ and Ca^2+^ in the water column. These water samples were stored in properly labelled preparation bottles at 4 °C and were used for subsequent Cd^2+^ and Ca^2+^ concentration analyses. The water samples (200 mL) were digested with 5 mL of a di-acid mixture (HNO_3_:HClO_4_ = 9:4) on a hot plate and filtered with a glass microfibre filter paper (Advantec Toyo) for the analysis of Cd^2+^ and Ca^2+^ using a flame atomic spectrophotometer (WFX-130A, Beijing Rayleigh Analytical Instruments Co., Ltd, China), according to the National Standard of China (GB 17378.4-2007, the section “seawater analysis” in “The speciation for marine monitoring”)^48^ at detection limits of 0.01 and 0.001 μg/L for Cd and Ca, respectively.

After exposure to Cd-contaminated seawater for 30 days at different *p*CO_2_ levels, five live individuals of each species were taken out for the Cd accumulation analysis. The individuals were dissected on ice, and the gills, mantle, and adductor muscles were peeled off and weighed separately. To obtain the dry mass, the different tissues were dehydrated in the oven to a constant weight at 75 °C. Dried tissues were first homogenized with a standard Teflon tissue homogenizer, followed by nitric acid digestion (1 g of each sample). Once the samples cooled down to room temperature, the sample digestions were filtered with a glass microfibre filter paper (Advantec Toyo) and diluted to 50 ml in volumetric flasks with deionized water. The concentrations of cadmium were then determined using a flame atomic spectrophotometer (WFX-130A), according to the National Standard of China (GB05009-15-2003)^49^ with a detection limit of 5 μg/kg. Three replicates were examined for each *p*CO_2_ level to obtain the average concentration. The Cd concentrations in the various tissues were then calculated and expressed in mg kg^−1^ dry weight. Similarly, after 30 days exposure, the entire soft body of *M. meretrix* was peeled off to determine Cd concentration (C) for THQ analysis. Cd concentrations in the whole soft body were then calculated and expressed in mg kg^−1^ wet weight.

Appropriate quality assurance procedures and precautions were carried out to ensure results reliability. Samples were carefully handled to avoid contamination, all the plastics and glasswares were cleaned by soaking in dilute HNO_3_ and then rinsed with distilled water prior to use, and reagents of analytical reagent grade were used. A standard reference materials (GBW08571) obtained from the National Research Center for Standard Reference Materials (Beijing China) was used in the analysis to ensure measurement accuracy. A recovery experiment was carried out by spiking the already analyzed sample and recoveries were found to be within ±5% of certified values.

### Expression analysis of pgp-5 gene

Total RNA of *T. granosa* was extracted from the gills, which were considered the main entry site for toxic metals in bivalves, with the RNAprep Pure Tissue Kit (Tiangen, DP431) according to the protocols provided by the manufacturer. RNA integrity was checked by gel electrophoresis and quantified spectrophotometrically with NanoDrop 1000 UV/visible spectrophotometer (Thermo Scientific). First strand cDNA was synthesized from high-quality total RNA using the M-MLV First Strand Kit (Invitrogen, C28025-032) following manufacturer’s instructions. Real-time quantitative PCR were conducted on the CFX96TM Real-Time System (Bio-Rad) in triplicates, in a total volume of 10 μL consisting of 5 μL of 2× Super Mix (Bio-Rad, 172-5201AP), 0.5 μL of each primer (10 μM), 1 μL of cDNA template, and 3 μL of double-distilled water. The following amplification protocol was used: 95 °C for 5 min, followed by 40 cycles (94 °C for 20 sec, 61 °C for 20 sec, and 72 °C for 20 sec). A melting curve analysis (MCA) was used to confirm the specificity and reliability of the PCR products. The 18S rRNA was employed as a reference for the calculation of the relative expression levels. The primers used are listed in Table 3 and all primers were synthesized by Sangon Biotech (Shanghai, China).

### Health risk assessment

The EDI of Cd was determined by the equation: EDI = (EF_r_ × E_D_ × MS × C)/(W_AB_ × T_A_), where EF_r_ is the exposure frequency^50^ (350 day/year); E_D_ is the exposure duration (70 years), which is equivalent to the average lifetime of adults^51^; MS is the average food meal size (21.43 g/person/day according to the dietary intake survey^52^); C is the obtained Cd concentration in the soft body of *M. meretrix*; W_AB_ represents the average body weight^53^, adults (70 kg); and T_A_ is the average exposure time for noncarcinogens (70 years × 365 day/year according to Wang *et al.*^54^). The THQ values of Cd through consumption of *M. meretrix* raised in different *p*CO_2_ levels were then estimated by the equation: THQ = EDI/RfD with the data obtained in the present study. Accoding to JECFA^55^, 0.83 × 10^−3^ μg/g/day was used as the oral intake reference dose (RfD).

### Statistics analysis

One-way analysis of variances (one-way ANOVAs) followed by post-hoc Tukey tests were performed to compare the Cd levels within various tissues, the Ca^2+^ and Cd^2+^ concentrations of the seawater, EDI and THQ values at different *p*CO_2_ levels. The analyses were performed using the “R” statistical software packages (R Development Core Team, 2012), T-tests were conducted to detect whether there was a significant alteration in the gene expression compared to that of the control. All of the data are presented as mean ± SD, and a *p*-value at *p* < 0.05 was taken as statistically.

## Additional Information

**How to cite this article**: Shi, W. *et al.* Ocean acidification increases cadmium accumulation in marine bivalves: a potential threat to seafood safety. *Sci. Rep.* **6**, 20197; doi: 10.1038/srep20197 (2016).

## Figures and Tables

**Table 1:** Ca^2+^ and Cd^2+^ (in mmol/L and mg/L) concentrations and Cd^2+^/Ca^2+^ in seawater after exposure to *p*CO_2_ trials.

pH	8.1	7.8	7.4
Ca^2+^ mmol L^−1^ (mg L^−1^)	9.14 ± 1.4E-4^a^ (365.6 ± 5.6E-3)	8.79 ± 1.5E-4^b^ (351.6 ± 6E-3)	7.82 ± 5.5E-5^c^ (312.8 ± 2.2E-3)
Cd^2+^ mmol L^−1^ (mg L^−1^)	3.8E-4 ± 3.6E-12^a^ (4.26E-2 ± 4.0E-10)	4.1E-4 ± 1.4E-11^b^ (4.59E-2 ± 1.6E-9)	4.4E-4 ± 1.2E-10^c^ (4.93E-2 ± 1.3E-8)
Cd^2+^/Ca^2+^	4.1E-5 ± 2.4E-14^a^ (1.17E-4 ± 6.8E-14)	4.7E-5 ± 2.5E-13^b^ (1.31E-4 ± 7.0E-13)	5.6E-5 ± 1.7E-12^c^ (1.58E-4 ± 4.8E-12)

The data were analysed by a one-way ANOVA, followed by post-hoc Tukey-test. Mean values that do not share the same superscript were significantly different.

**Table 2:** The estimated Cd EDIs and THQs of *M. meretrix* after 30 days exposure to Cd at different *p*CO_2_ levels.

pH	8.1	7.8	7.4
C (mg/kg, wet weight)	0.55 ± 4.3E-6^a^	0.65 ± 1.7E-6^b^	0.70 ± 1.5E-6^c^
EDI (μg/g/day)	0.16E-3 ± 3.7E-13^a^	0.19E-3 ± 1.4E-13^b^	0.21E-3 ± 1.3E-13^c^
THQ	0.19 ± 5.4E-7^a^	0.23 ± 2.1E-7^b^	0.25 ± 1.9E-7^c^

The data were analysed by a one-way ANOVA, followed by post-hoc Tukey-test. Mean values that do not share the same superscript were significantly different.

**Table 3:** Primers sequences of genes used in real-time PCR analysis.

Gene	Primer sequence (5′ to 3′)	Accession no.
*pgp-5:* F	TAGGCGTGGCATAGTAGAT	JZ875856
*pgp-5:* R	CTTATTGGCATCGTGTCTTG	
*18s rRNA:* F	CTTTCAAATGTCTGCCCTATCAACT	JN974506.1
*18s rRNA:* R	TCCCGTATTGTTATTTTTCGTCACT	
